# Malaria‐Associated Acute Kidney Injury in a Low‐Endemic Region: A Case Series of Five Imported *Plasmodium falciparum* Infections

**DOI:** 10.1155/crdi/5583026

**Published:** 2026-01-31

**Authors:** Daniel P. Mujuni, Abid M. Sadiq, Elisha Luhwago, Datius Mutalemwa, Abel Mwanga, Leanji Leonard, Elifuraha W. Mkwizu, Elichilia R. Shao, Kajiru G. Kilonzo

**Affiliations:** ^1^ Department of Internal Medicine, KCMC University, Moshi, Tanzania, kcmuco.ac.tz; ^2^ Department of Internal Medicine, Kilimanjaro Christian Medical Center, Moshi, Tanzania, kcmc.ac.tz

**Keywords:** clinical presentation, hemodialysis, low-endemic regions–imported malaria, malaria-associated acute kidney injury, pathophysiology, *Plasmodium falciparum*

## Abstract

Malaria‐associated acute kidney injury (MAKI) is a formidable and potentially fatal complication of malaria. Early recognition and timely management can significantly reduce morbidity and mortality, particularly in resource‐limited settings. This case series describes five patients with imported *Plasmodium falciparum* complicated by MAKI who were treated at Kilimanjaro Christian Medical Center (KCMC) in northeastern Tanzania between December 2023 and January 2024. The cohort comprised of four males and one female, with a mean age of 38.2 ± 18.6 years. All patients presented with a history of fever and a recent history of travel to high‐endemic regions. The mean serum creatinine and urea levels at admission were 736 ± 346.4 μmol/L and 23.1 ± 14.1 mmol/L, respectively. Four patients required hemodialysis, and one experienced malaria recrudescence. Overall survival was 100%. This case series highlights pathophysiological mechanisms, clinical presentations, diagnostic challenges, and management outcomes of MAKI, illustrating its modified natural history. Despite advances in understanding MAKI, early diagnostic and treatment challenges persist. This case series aimed to elucidate the underrecognized burden and patterns of MAKI in a resource‐limited, low‐endemic region within an endemic country.

**Key Clinical Message**
 MAKI should be suspected in patients presenting with acute kidney injury and recent travel to endemic regions. Early diagnosis and timely initiation of renal replacement therapy are critical to improving outcomes and reducing mortality.

## 1. Introduction

Malaria is a protozoan infection caused by *Plasmodium falciparum, vivax, malariae, ovale, and knowlesi* and is transmitted by the female *Anopheles* mosquito. Among these species, *Plasmodium falciparum* is most commonly associated with severe disease. Malaria remains a significant global health burden, with an estimated 249 million cases reported worldwide in 2022, of which approximately 94% occurred in Sub‐Saharan Africa and other tropical regions. Severe malaria accounts for nearly two million cases annually and contributes to approximately 608,000 deaths globally [1].

**FIGURE 1:**
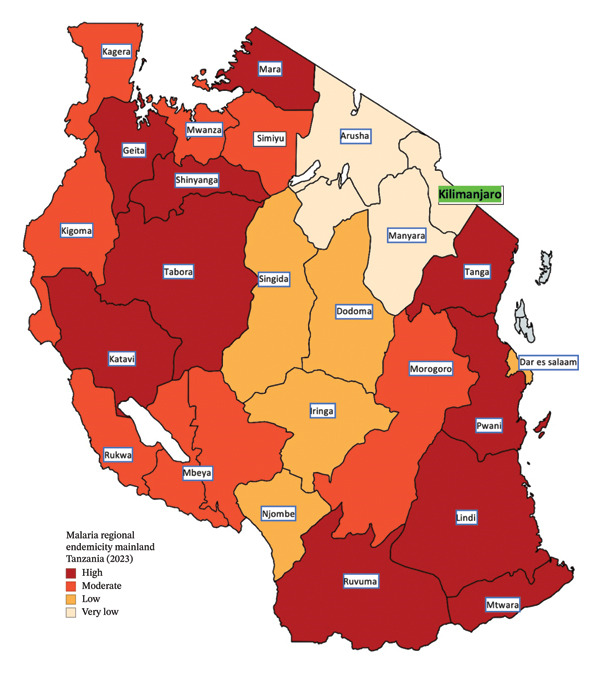
Malaria endemicity regional stratification of mainland Tanzania. High endemicity regions (deep red on the map) are in the south, northwest, and coastal regions, and very low endemicity regions (cream on the map) are in high‐altitude northeastern regions [2].

In Tanzania, malaria prevalence is estimated at 9%, but it is unevenly distributed across the country. Coastal and low‐altitude regions report prevalence rates of up to 15%, whereas high‐altitude, low‐endemic regions report rates below 1% [2] (Figure 1). Population movements contribute to the importation of malaria into the low‐endemic regions. Among nonimmune travelers infected with *P. falciparum* from endemic regions, acute kidney injury (AKI) has been reported in 34%–52% of cases [3].

Malaria typically presents as a febrile illness with nonspecific clinical symptoms; however, Malaria‐associated AKI (MAKI) is a serious but often underrecognized complication. In endemic regions, MAKI occurs in up to 40% of adult patients with severe *P. falciparum* malaria and is associated with mortality rates as high as 75% when renal replacement therapy (RRT) is delayed or unavailable [4].

MAKI arises through diverse pathophysiological mechanisms, including direct parasite‐induced injury, immune‐mediated response, microvascular dysfunction, and hemodynamic alterations [5]. Clinically, MAKI ranges from mild renal impairment to severe, life‐threatening AKI, necessitating prompt recognition and intervention. Diagnosis relies on clinical assessment supported by laboratory and imaging findings, although it is often complicated by overlapping malaria‐related organ dysfunction [6]. Management includes prompt antimalarial therapy, supportive care, and timely RRT in severe cases [5].

MAKI poses significant diagnostic and management challenges, particularly in a low‐endemic, resource‐limited setting. A thorough understanding of its pathophysiology and clinical presentation is essential for timely recognition and intervention. MAKI represents a medical emergency characterized by multisystem involvement and may manifest differently in adults and children. Nevertheless, recent studies indicate that cerebral involvement, renal impairment, and acidosis are independent predictors of mortality across all age groups [5].

AKI in severe *falciparum* malaria is primarily caused by acute tubular necrosis (ATN) and is defined by a serum creatinine > 265 μmol/L or serum urea > 20 mmol/L [3]. AKI develops in up to 40% of both adult and pediatric patients with severe malaria [3]. However, the World Health Organization (WHO) criteria may inadequately define AKI in severe *falciparum* malaria, potentially leading to underestimation of its true incidence. The Kidney Disease Improving Global Outcomes (KDIGO) classification provides a more standardized and dynamic definition, incorporating changes in estimated glomerular filtration rate (eGFR) and/or urine output, and is now widely adopted in both clinical and research setting [7]. In this case series, we present five patients with MAKI, describing their clinical presentation, course, and successful treatment outcomes.

## 2. Case Presentation

### 2.1. Case 1

An 18‐year‐old male presented at our facility with a 32‐h history of altered consciousness. Nine days prior, shortly after returning home to Kilimanjaro from boarding school in Tanga, he developed intermittent fever with rigors and chills, headache, and joint and muscle aches. Two days later, he developed nausea, nonprojectile vomiting, abdominal pain, and generalized body malaise. He was taken to a district hospital, where, upon arrival, he had a tonic–clonic seizure lasting 2 min, followed by unresponsiveness, without fecal or urine incontinence.

He was managed as a case of bacterial meningitis. After little to no improvement two days later, he was diagnosed with severe malaria (994P/200WBC) and AKI with serum creatinine (sCr) of 272.54 μmol/L and urea of 10.6 mmol/L. He received two doses of intravenous (IV) artesunate and supportive care before being referred to our facility for further management.

On his examination, he had a Glasgow Coma Scale (GCS) of 7/15 (E2V3M2). He was febrile; he had a urine catheter in situ with 100 mL of cola‐colored urine. His vitals were as follows: blood pressure (BP) of 148/72 mmHg, temperature (T) of 37.4°C, pulse rate (PR) of 106 bpm, respiratory rate (RR) of 15 cpm, oxygen saturation (SpO_2_) of 97% on room air (RA), and random blood glucose (RBG) of 5.8 mmol/L. Pupils were equal, central, symmetrical, and reactive to light. Fundoscopy showed no papilledema, and there were no signs of meningeal irritation. Examination of other systems was unremarkable.

#### 2.1.1. Investigations and Clinical Course

On admission, his full blood picture (FBP) revealed a white blood cell (WBC) count of 3.92 × 10^9^/L, hemoglobin (Hb) of 12.4 g/dL (normocytic normochromic), and platelet (PLT) count of 77 × 10^9^/L, and sCr of 152 μmol/L, urea of 11.35 mmol/L, aspartate aminotransferase (AST) of 160.12 U/L, alanine aminotransferase (ALT) of 61.63 U/L. The malaria rapid diagnostic test (MRDT) was positive, with a malaria blood smear (BS) showing 76 parasites/200WBC. Lumbar puncture (LP) was unremarkable. A diagnosis of MAKI and cerebral malaria was established.

He received IV artesunate 150 mg at 0, 12, and 24 h and IV crystalloids (1 L bolus followed by 2 L maintenance for 24 h). On the 2^nd^ day, his BS showed 25P/200WBC, sCr of 134 μmol/L, and urea of 12.24 mmol/L. Urinalysis revealed turbid and dark urine, with protein 1+, blood 3+, numerous WBC and RBC per high‐power field (hpf), epithelial cells 2+, and epithelial cast 1+.

At the 24^th^ hour, his GCS improved to 14/15 (E4V4M6), and his urine output reached 1140 mL over 12 h. He was switched to oral artemether + lumefantrine (ALU) 140 mg daily for 3 days, with a fluid intake of 3.5 L/24 h. On the 3^rd^ day, his urine output further improved to 2090 mL/24 h. He was discharged on Day 5 and scheduled for follow‐up in 2 weeks (see Tables 1 and 2).

**TABLE 1:** Malaria parasitemia trend (*N* = 5).

Cases	Malaria blood slide (parasites/200 WBC)			
	On admission	Day 2	Day 3	At 2 weeks
1	76	25	No parasite	412
2	No parasite	No parasite	—	—
3	32	No parasite	—	—
4	No parasite	89	No parasite	—
5	803	No parasite	—	—

Abbreviation: WBC, white blood cell.

**TABLE 2:** Trends of serum creatinine levels in μmol/L over time (*N* = 5).

Cases	Admission	Day 2‐3	Day 5–7	Day 10–14	2 weeks postdischarge	1 month postdischarge
1	152	116	—	—	68	—
2	979	484	312	221	91	—
3	1011	585	703	415	261	144
4	738	639	1134	427	198	78
5	800	635	589	300	89	—

#### 2.1.2. Follow‐Up and Outcome

At follow‐up, he presented with a 4‐day history of fever, headache, nausea, vomiting of bilious material, and body malaise. BS showed 412P/200WBC, suggesting recrudescent malaria, and he was restarted on artesunate combined therapy. We managed to contact him 6 months later, and he reported full recovery and had resumed his studies.

### 2.2. Case 2

A 34‐year‐old male from Hai district, Kilimanjaro, was referred to our facility with a 1‐week history of generalized throbbing headaches, intermittent high‐grade fevers, muscle and joint pain, and easy fatigability. Prior to hospital presentation, he had self‐medicated with sulfamethoxypyrazine/pyrimethamine for malaria without improvement and decided to seek medical assistance at the district hospital. He was diagnosed with malaria and started on antimalarial treatment IV artesunate; his renal function was not assessed.

Despite therapy, his condition deteriorated, with the onset of nausea, vomiting, epigastric pain, and bilateral ankle swelling. He also reported oliguria for the preceding 4 days. Notably, he had traveled to Dar es Salaam, a malaria‐endemic region, 2 weeks prior to symptom onset.

On his examination, he was conscious with facial puffiness, conjunctival pallor, and Grade 2 bilateral lower limb pitting edema. He was afebrile to the touch. His vitals were as follows: BP of 135/66 mmHg, T of 36.1°C, PR of 71 bpm, RR of 19 cpm, SpO_2_ of 98% on RA, and RBG of 5.5 mmol/L. He had a symmetrically distended abdomen with normal contours, epigastric tenderness, and positive shifting dullness. The rest of the examination was unremarkable.

#### 2.2.1. Investigations and Clinical Course

Investigations on admission revealed Hb of 9.4 g/dL (normocytic normochromic), PLT count of 74 × 10^9^/L with sCr of 979 μmol/L, urea of 31.84 mmol/L, and Na^+^ of 110.34 mmol/L. MRDT was positive, though BS showed no malaria parasite. A diagnosis of MAKI was established.

He received IV furosemide 120 mg stat and IV pantoprazole 40 mg q24 h for gastritis. Initial urine output was 70 mL after 2 h, and signs of fluid overload persisted. Hemodialysis (HD) was initiated on the 2^nd^ day, with sessions lasting 2–3.5 h. The initial three sessions were performed on consecutive days. For the first session, the duration was strictly 2 h, with dialysate sodium set at 125–130 mmol/L and 2–2.5 L of ultrafiltration during the first two sessions due to dilutional hyponatremia and fluid overload. By the third session, urine output increased to 600 mL/24 h, serum sodium improved to 132.13 mmol/L, and the patient continued with intermittent HD every other day. On the 7^th^ day, he developed a fever (T of 37.4 °C); twin cultures were collected, and empirical IV vancomycin 1 g q48 h and IV ciprofloxacin 400 mg q24 h were started for presumed catheter‐related bloodstream infection.

By the 11^th^ day, after 5 HD sessions, his urine output had normalized to 2000 mL/24 h; lower limb edema, facial puffiness, and ascites had resolved; and cultures showed no bacterial growth. He had significant improvement, and at Day 13, he was discharged with follow‐up scheduled at the nephrology clinic (see Tables 1 and 2).

#### 2.2.2. Follow‐Up and Outcome

At 2 weeks follow‐up, he was stable and asymptomatic, with sCr of 91 μmol/L.

### 2.3. Case 3

A 51‐year‐old male from Moshi municipality, Kilimanjaro, presented with a 5‐day history of headaches, low‐grade intermittent fever, body malaise, loss of appetite, nausea, and vomiting. He was initially seen at a local health center, diagnosed with malaria, and discharged on oral ALU. However, his symptoms persisted, evolving to include painful urination, urgency, reduced urine output, and a sensation of incomplete bladder emptying. He returned to the health facility on the 4^th^ day of his illness, and he was referred to our facility for further management. The patient had recently returned from a 2‐week work trip to Handeni District in Tanga, a known malaria‐endemic area.

On his examination, he was alert and oriented, afebrile, with conjunctival pallor and scleral icterus. His vitals were BP of 132/65 mmHg, T of 36.5°C, PR of 71 bpm, RR of 20 cpm, SpO_2_ of 97% on RA, RBG of 5.5 mmol/L, and a urine catheter in situ with 230 mL of amber‐colored urine. Abdominal exam revealed epigastric tenderness, while other systems were unremarkable.

#### 2.3.1. Investigations and Clinical Course

On admission, MRDT was positive, BS showed 32P/200WBC, a WBC count of 16.21 × 10^9^/L with neutrophilia, Hb of 7.3 g/dL (microcytic normochromic), PLT count of 63 × 10^9^/L, ESR of 69 mm/1 h, sCr of 1011 μmol/L, urea of 13.2 mmol/L, AST of 99.25 U/L, ALT of 31.13 U/L, and bilirubin (total 167.4 μmol/L; direct 158.24 μmol/L). Urinalysis showed protein traces, blood 2+, RBC 5‐10/hpf, and bacteria 2+. The diagnoses of MAKI and UTI were made. He was treated with IV artesunate 180 mg at 0, 12, and 24 h; had septic workups; and empirically started on IV ceftriaxone 1 g q12 h, and the urine catheter was removed. Concurrently, he was initiated on HD, with sessions lasting 2–3.5 h. The initial three sessions were performed on consecutive days and thereafter every other day. By the 3^rd^ day, he had urine output of 1000 mL/24 h, urea of 19.09 mmol/L, and malaria BS was negative. On the 6^th^ day despite persistently elevated sCr, he was clinically stable, and he was discharged on the 14^th^ day (see Tables 1 and 2).

#### 2.3.2. Follow‐Up and Outcome

After 5 HD sessions, sCr improved to 261 μmol/L at 2‐week follow‐up postdischarge, and it normalized by 3 months, with the patient remaining well.

### 2.4. Case 4

A 25‐year‐old male, a referral from Arusha, presented with a one‐week fever and altered mental status for one day. This illness began as a low‐to high‐grade fever with rigors, chills, and malaise. He was discovered unconscious in his room a day before admission, resuscitated at a local hospital, but he remained verbally unresponsive. He also had anuria for 2 days. He was subsequently referred to our facility for further management. Notably, he had recently traveled to Tanga, shortly after his return to Arusha, he was diagnosed and treated for malaria as an outpatient, but did not complete his antimalarial course.

On admission, he was drowsy with a GCS of 14/15 (E4V4M6). His vitals were PR of 62 bpm, BP of 121/53 mmHg, T of 36°C, SpO_2_ of 98% on RA, RR of 22 cpm, and RBG of 6.5 mmol/L. His central nervous system illustrated a reduced GCS but, no signs of meningeal irritation, and the rest of the examination was unremarkable.

After catheterization, 100 mL of cola‐colored urine was collected; he remained anuric for > 12 h.

#### 2.4.1. Investigations and Clinical Course

On admission, MRDT was positive, malaria BS showed no parasites, and LP was unremarkable. FBP showed Hb of 9.4 g/dL, PLT count of 119 × 10^9^/L, sCr of 738 μmol/L, urea of 15.38 mmol/L, and Na^+^ of 132.51 mmol/L. Urinalysis showed turbid dark urine with protein 3+, no blood, SG 1.015, leucocytes 1+, numerous WBC/hpf, RBC 20–25/hpf, bacteria 3+, and WBC casts.

He was reinitiated on a full course of antimalarial with IV artesunate 144 mg at 0, 12, and 24 h. He also received IV ceftriaxone 1 gm q12 h for suspected UTI, and HD was initiated the same day. On the 2^nd^ day, he started oral ALU for 3 days, and repeat malaria BS showed 89P/200WBC. On the 3^rd^ day, his blood culture yielded methicillin*-*resistant *Staphylococcus aureus* (MRSA) sensitive to vancomycin; he was thereafter switched to IV vancomycin 1 g q48 h.

By the end of the first week, he had stabilized clinically, was asymptomatic, and had normal cognition with adequate urine output after 7 HD sessions. He was discharged on Day 17 (see Tables 1 and 2).

#### 2.4.2. Follow‐Up and Outcome

At the 2‐week follow‐up, the patient remained clinically stable. A subsequent review one month later revealed normalized renal function with sCr of 78 μmol/L.

### 2.5. Case 5

A 63‐year‐old female was referred to our facility with a 3‐day history of progressively worsening altered mental status. Initially, she exhibited a reduced attention span and disorganized speech, which progressed to verbal unresponsiveness and uncooperative behavior. Five days prior to the onset of confusion, she developed a high‐grade fever, joint and muscle pain, loss of appetite, nausea, vomiting, and scleral icterus.

She was admitted to a peripheral hospital where, due to suspicion of meningitis, she received IV ceftriaxone 2 gm every 12 h. During the course of her treatment, she developed oliguria with dark, amber–cola–colored urine. Laboratory investigations showed renal impairment, prompting referral to our facility for further management. Of note, the patient had traveled to Dar es Salaam one week prior to symptom onset.

On examination, she was drowsy, afebrile to the touch, and exhibited scleral icterus. Her vitals were BP of 111/65 mmHg. PR of 85 bpm, T of 36.8°C, SpO_2_ of 98% in RA, and RR of 16 cpm. A urinary catheter was in situ, draining 300 mL of cola‐colored urine. On her central nervous exam, she had a reduced GCS of 14/15 (E4V4M6), with impaired cognitive function, specifically in attention and concentration, and no signs of meningeal irritation, and the rest of the examination was unremarkable.

#### 2.5.1. Investigations and Clinical Course

On admission, MRDT was positive, and malaria BS showed 803P/200WBC. FBP revealed Hb of 9.3 g/dL, PLT count of 53 × 10^9^/L, ESR of 84 mm/1 h, sCr of 800 μmol/L, urea of 43.57 mmol/L, Na^+^ of 127.87 mmol/L, K^+^ of 6.08 mmol/L, AST of 100.27 U/L, ALT of 43.21 U/L, and bilirubin (total 389 μmol/L; direct 321.8 μmol/L). MAKI was diagnosed; she received IV artesunate followed by ALU, and HD was immediately initiated. On the 2^nd^ day, her BS for malaria was negative.

Her condition improved steadily, with a urine output of 1200 mL/24 h on the 7^th^ day and between 1300 and 1700 mL/24 h from the 12^th^ day onward, accompanied with resolution of symptoms (see Tables 1 and 2).

#### 2.5.2. Follow‐Up and Outcome

**FIGURE 2:**
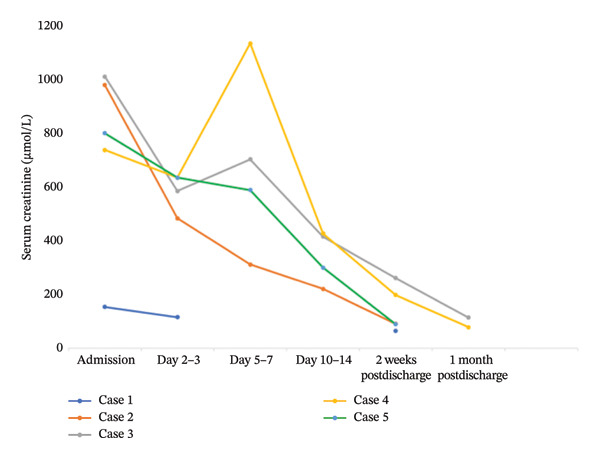
Serum creatinine trends over time.

The patient underwent a total of seven HD sessions and was discharged in a stable condition with a follow‐up plan. At her 2‐week nephrology clinic visit, she remained clinically stable (Figure 2).

## 3. Discussion

The presented cases highlight the clinical presentation, diagnosis, pathogenesis, and successful management of MAKI in low‐endemic, resource‐limited setting, demonstrating favorable outcomes despite its typical high mortality.

In the literature, the clinical presentation of MAKI has a multitude of clinical symptoms with multiorgan involvement; the most common include fever, headache, and reduced urine output. Amongst our patients, they presented with fever, headache, reduced urine output, and multiorgan involvement; two had cerebral malaria, and two had raised transaminases, implying hepatic involvement [8]. One case had dipstick proteinuria of 3+, indicating heavy proteinuria without nephrotic syndrome and rarely patients have been reported to present with nephrotic syndrome in *P. falciparum* [3, 9, 10]. The clinical presentation of MAKI appears to be elusive, but a hallmark of complicated malaria; two of the patients (Cases 2 and 3) presented with mild symptoms of malaria to warrant outpatient management, only to develop life‐threatening AKI, which was initially missed.

Due to this, clinicians would be wise to have a longer patient follow‐up approach and shy away from a one‐touch approach in the management of malaria amongst patients in low‐endemicity areas, as they have an increased risk of developing severe malaria with sustained complications due to low immunity to falciparum [3]. Moreover, one of the cases had recrudescent malaria due to hyperparasitemia and possibly artesunate resistance [11], and another took 3 months for kidney function to normalize.

The diagnosis of MAKI was made based on both MRDT and BS for malaria [12], alongside urine output assessment and elevated creatinine [7]. Moreover, a follow‐up BS was performed in all patients to ensure parasite clearance. This proved to be crucial, as one patient’s BS revealed the presence of malaria parasites 24 h postartemisinin therapy; this could be explained by BS being an operator‐dependent investigation.

Over the past three decades, MAKI has been more prevalent than when severe malaria was complicated by cerebral involvement [13]. Clearly, even with low‐density parasitemia, all patients presented with the diagnosis of MAKI, and 4 out of 5 patients required RRT. This could be attributed to the lack of protective immunity against *P. falciparum* amongst individuals residing in nonendemic regions who traveled to high‐endemic areas. Studies have shown nonimmunized or partially immunized individuals have an increased risk of complicated malaria, including MAKI [3]. Other complementary findings, including proteinuria, microalbuminuria, and urinary casts, have been reported in 20%–50% of cases [10].

The pathogenesis of MAKI remains obscure. However, one of the proposed mechanisms involves blockage of renal microcirculation due to sequestration of *Plasmodium falciparum*–infected erythrocytes. This is mediated by *Plasmodium falciparum*–derived erythrocyte membrane protein 1 (pfEMP1), which binds to receptors such as CD‐36 and the endothelial protein C receptor (EPCR), as well as to uninfected erythrocytes in the renal vasculature and other organs [14]. Second, immune‐mediated glomerular injury through proinflammatory mediators such as TNF‐α, IFN‐γ, and IL‐1*α*, 6, and 8; reactive oxygen species; Th_1_ cell– and Th_2_ cell–mediated immunity; and activation of the alternative complement pathway. Lastly, volume depletion due to reduced fluid intake and fluid losses from vomiting and fever is another proposed mechanism [14]. Histopathologically, ATN is the most common observed lesion in MAKI, while interstitial nephritis and glomerulonephritis are reported less frequently [10].

On further examination of the cohort, all patients who had a low PLT count and Hb level less than 10 g/dL required RRT [4]. This is in keeping with existing literature, which has shown an association of MAKI with thrombocytopenia, anemia, male sex, and age [10, 15].

Treatment of MAKI is centered on early and prompt initiation of parenteral antimalarial drugs, careful fluid and electrolyte management, and initiation of RRT when necessary [3]. Timely initiation of RRT in patients presenting with Stage 3 AKI was associated with improved clinical outcome including complete recovery of kidney function. The benefits of RRT began to appear for most patients after the 3^rd^ HD session, and one case recovered without HD (Case 1). None of the patients had a known prior history of kidney disease; however, this assessment was limited by the absence of baseline serum creatinine measurements. All patients had partial to complete recovery at discharge. At two weeks postdischarge, four of the five patients had fully recovered from the renal injury. Overall, the mean time to full recovery was 30± 31 days, with one patient representing an outlier. Despite these favorable short‐term outcomes, the long‐term prognosis of MAKI remains uncertain, as studies have shown that patients who developed Stage 3 AKI have more than a 20‐fold increased risk of developing chronic kidney disease (CKD) later on as compared to individuals who never had AKI [16]. Moreover, MAKI without any form of treatment is a certain death. A meta‐analysis by Olowu et al. in Sub‐Saharan Africa showed that for those who needed HD but could not access it, the mortality was above 80% [17].

The above observation seems to be in contrast to the Rotterdam cases of imported malaria, in which most of the patients with severe malaria had recovered from the renal insult without RRT; furthermore, the return of creatinine to baseline was 17 ± 6 days [3], 13 days shorter in comparison to our 5 cases. This can be attributed to early detection and diagnosis of MAKI, in contrast to our setting, where the patient had a late diagnosis with Stage 3 AKI.

## 4. Conclusion

We report five cases of MAKI; four of which required RRT. In low‐endemic areas, clinicians must maintain a high index of suspicion for MAKI, particularly in patients with a travel history to high‐endemic regions. The diagnosis of MAKI should incorporate both MRDT and confirmatory blood microscopy, alongside AKI classification based on KDIGO criteria to facilitate early detection. These cases underscore the need for heightened awareness of imported severe malaria due to *P. falciparum*, which may present atypically and require a longer patient follow‐up. Early recognition and timely initiation of RRT are critical for reducing morbidity and mortality in MAKI.

## Author Contributions

Daniel P. Mujuni, Kajiru G. Kilonzo, and Elichilia R. Shao were principal investigators. Abid M. Sadiq and Daniel P. Mujuni contributed to the design of the study. Abel Mwanga, Elisha Luhwago, Datius Mutalemwa, Leanji Leonard, and Daniel P. Mujuni performed the data collection of the cases. Abid M. Sadiq, Kajiru G. Kilonzo, Elichilia R. Shao, and Daniel P. Mujuni drafted the manuscript. Leanji Leonard, Kajiru G. Kilonzo, Daniel P. Mujuni, and Abid M. Sadiq participated in editing the manuscript. Kajiru G. Kilonzo, Daniel P. Mujuni, Elifuraha W. Mkwizu, Elichilia R. Shao, and Abid M. Sadiq edited the final draft of the manuscript. Daniel P. Mujuni, Kajiru G. Kilonzo, Elichilia R. Shao, Elifuraha W. Mkwizu, Abel Mwanga, Datius Mutalemwa, Leanji Leonard, and Elisha Luhwago were responsible for patient care and management.

## Funding

The authors received no financial support for research, authorship, and/or publication of this article.

## Ethics Statement

Our institution does not require ethical approval for reporting individual cases or case series.

## Consent

Written informed consent was obtained from the patients for their anonymized information to be published in this article.

## Conflicts of Interest

The authors declare no conflicts of interest.

## Data Availability

The data that support the findings of this study are openly available in figshare at https://figshare.com/s/f8ff5e732e14d939ba57.

## References

[1] World Health Organization , World Malaria Report 2022, 2022.

[2] U.S. President’s malaria initiative , U.S. President’s Malaria Initiative Tanzania Mainland Malaria Operational Plan FY 2022, 2022.

[3] Koopmans L. C. , Van Wolfswinkel M. E. , Hesselink D. A. et al., Acute Kidney Injury in Imported Plasmodium falciparum Malaria, Malaria Journal. (2015) 14, no. 1, 10.1186/s12936-015-1057-9, 2-s2.0-84951267366. PMC4690233 26702815

[4] Plewes K. , Royakkers A. A. , Hanson J. et al., Correlation of Biomarkers for Parasite Burden and Immune Activation with Acute Kidney Injury in Severe Falciparum Malaria, Malaria Journal. (2014) 13, no. 1, 10.1186/1475-2875-13-91, 2-s2.0-84899060619. PMC3995633 24618154

[5] Plewes K. , Turner G. D. H. , and Dondorp A. M. , Pathophysiology, Clinical Presentation, and Treatment of Coma and Acute Kidney Injury Complicating Falciparum Malaria, Current Opinion in Infectious Diseases. (2018) 31, no. 1, 69–77, 10.1097/QCO.0000000000000419, 2-s2.0-85041453120. 29206655 PMC5768231

[6] Kazinga C. , Bednarski O. , Aujo J. C. et al., Acute Kidney Injury in Severe Malaria: a Serious Complication Driven by Hemolysis, Seminars in Nephrology. (2025) 45, no. 3, 10.1016/j.semnephrol.2025.151614. 40410006

[7] Kellum J. A. , Lameire N. , Aspelin P. et al., Kidney Disease: Improving Global Outcomes (KDIGO) Acute Kidney Injury Work Group. KDIGO Clinical Practice Guideline for Acute Kidney Injury, Kidney International Supplements. (2011) 2012, no. 2, 1–138, 10.1038/kisup.2012.1, 2-s2.0-84884342549.

[8] Naik H. , Acharya A. , and Rout S. , Clinical Profile and Treatment Outcomes of Patients with Malaria Complicated by Acute Kidney Injury, Saudi Journal Kidney Displacement Transplation. (2023) 34, no. 2, 117–124, 10.4103/1319-2442.391889. 38146720

[9] Kurnianingrum N. M. A. and Ayu N. P. , Severe Falciparum Malaria with Acute Kidney Injury: a Case Report, IOP Conference Series: Materials Science and Engineering. (2018) 434, 10.1088/1757-899X/434/1/012316, 2-s2.0-85058299650.

[10] Da Silva Junior G. B. , Pinto J. R. , Barros E. J. G. , Farias G. M. N. , and Daher E. D. F. , Kidney Involvement in Malaria: An Update, Revista do Instituto de Medicina Tropical de Sao Paulo. (2017) 59, 10.1590/S1678-9946201759053, 2-s2.0-85026828903. PMC5626226 28793022

[11] Landre S. , Bienvenu A. L. , Miailhes P. et al., Recrudescence of a High Parasitaemia, Severe Plasmodium falciparum Malaria Episode, Treated by Artesunate Monotherapy, International Journal of Infectious Diseases. (2021) 105, 345–348, 10.1016/j.ijid.2021.02.080. 33636354

[12] Omary M. , Abdallah L. , Sigsbert M. , and Andrea B. , Malaria Diagnosis, Treatment and Preventive Therapies 2020 National Malaria Control Programme National Guidelines for the United Republic of Tanzania Ministry of Health, Community Development, Gender, Elderly and Children National Malaria Control Programme. (2020) .

[13] White N. J. , Severe Malaria, Malaria Journal. (2022) 21, no. 1, 10.1186/s12936-022-04301-8. PMC9536054 36203155

[14] Chellappan A. and Bhadauria D. S. , Acute Kidney Injury in Malaria: An Update, Clinical Queries: Nephrology. (2016) 5, no. 1, 26–32, 10.1016/j.cqn.2016.04.004.

[15] Muhamedhussein M. S. , Ghosh S. , Khanbhai K. , Maganga E. , Nagri Z. , and Manji M. , Prevalence and Factors Associated with Acute Kidney Injury Among Malaria Patients in Dar es Salaam: a Cross-Sectional Study, Malar Residency Treatment. (2019) 2019, 1–7, 10.1155/2019/4396108. PMC6702846 31485321

[16] Coca S. G. , Singanamala S. , and Parikh C. R. , Chronic Kidney Disease After Acute Kidney Injury: a Systematic Review and meta-analysis, Kidney International. (2012) 81, no. 5, 442–448, 10.1038/ki.2011.379, 2-s2.0-84857112728. 22113526 PMC3788581

[17] Olowu W. A. , Niang A. , Osafo C. et al., Outcomes of Acute Kidney Injury in Children and Adults in sub-Saharan Africa: a Systematic Review, Lancet Global Health. (2016) 4, e242–e250, 10.1016/S2214-109X(15)00322-8, 2-s2.0-84962600421. 27013312

